# Effect of hydrothermal aging on color stability and translucency of two zirconia generations compared to lithium disilicate ceramics

**DOI:** 10.34172/joddd.41227

**Published:** 2024-09-07

**Authors:** Atef Ahmed Elzoughary, Tamer Abel Rahim Hamza, Mohamed Fawzy Metwally

**Affiliations:** Department of Crown and Bridge, Faculty of Dental Medicine, Al-Azhar University, Cairo, Egypt

**Keywords:** Aging, Ceramics, Color, Lithium disilicate, Tooth discoloration, Zirconium oxide

## Abstract

**Background.:**

An esthetically acceptable ceramic restoration should have optical properties like the teeth and reflect, transmit, and absorb light. The present investigation compared how hydrothermal aging affected the properties of two types of zirconia and lithium disilicate.

**Methods.:**

Thirty rectangular samples (12×14×1 mm) were prepared and sectioned from three different ceramic blocks/blanks (n=10), then assigned into three groups according to the ceramic type: group Z: IPS e.max ZirCAD prime, gradient zirconia (3Y/5Y-TZP); group K: Katana UTML (5Y-TZP); and group E: IPS e.max CAD (lithium disilicate). Color analysis of samples was performed before and after hydrothermal aging (1, 3, and 5 hours) using a spectrophotometer. Color difference (∆E_00_), translucency parameter (TP_00_), and contrast ratio (CR) were evaluated. The microstructural analysis was performed using x-ray diffraction (XRD). Data were statistically analyzed at a significance level of *P*<0.05.

**Results.:**

A statistically significant variation was observed across means of ∆E_00_, TP_00_, and CR at different times. Group Z displayed the highest statistically significant mean ∆E_00_. Group E demonstrated the greatest statistically significant mean TP_00_. Group K exhibited the most statistically significant mean CR.

**Conclusion.:**

Hydrothermal aging significantly affected the optical characteristics of lithium disilicate and zirconia ceramics. The translucency of samples increased with aging.

## Introduction

 Due to the growing need for naturally appearing restorations, the superior aesthetics and biocompatibility of ceramic restorations are the critical reasons for their increasing popularity.^1^ Different ceramic systems, including lithium disilicate, zirconium oxide, and derivatives, have been introduced to dentistry to meet these demands.^2-5^ Lithium disilicate is suitable for esthetic rehabilitation because of its unique microstructure, which is essential to its optical and mechanical characteristics.^6-9^ However, to combine aesthetics with superior strength properties, the manufacturers of dental materials have produced highly translucent monolithic zirconia.^10^

 Zirconia is a polycrystalline ceramic that is devoid of glass content. It is polymorphic in nature, meaning it can exist in three different shapes: cubic, tetragonal, and monoclinic. Pure zirconia exists in a monoclinic shape under ambient temperature, transforming to tetragonal at 1170 °C and cubic at 2370 °C. Volumetric change (approximately a 5% increase) coincides with the transition from monoclinic to tetragonal shape.^11^ The inclusion of stabilizing oxides, including yttrium, cerium, magnesium, and calcium oxides, helps stabilize zirconia in its tetragonal and cubic phases under ambient temperature.^12^ Yttrium oxide is the most commonly used stabilizer, where yttria-stabilized tetragonal zirconia polycrystalline (Y-TZP) was demonstrated to have the most pronounced phase transformation toughening.^13^ Using 3 mol% yttria to stabilize zirconia (3Y-TZP) is the initially produced Y-TZP in dentistry. One drawback of this generation is its whitish opaque color; therefore, veneering porcelain was applied to the zirconia core to maximize its aesthetic possibility. The main problem encountered with zirconia-based restorations is the chipping of the veneering porcelain.^14^ In response to this issue, monolithic translucent zirconia was introduced to allow their use in a fully contoured condition.^15,16^

 Several modifications were added to make zirconia suitable for monolithic restorations with adequate esthetics for anterior or posterior restorations. As part of this, the yttria concentration was raised from 3 mol% (3Y-TZP) to 4 mol% (4Y-TZP) and 5 mol% (5Y-TZP), in addition to introducing the polychromatic zirconia (multilayered).^15,16^ Another multilayered technique has recently been released, combining many zirconia generations into one blank to get the benefits of zirconia that is both conventional and ultra-translucent by combining the strong translucency of 5Y-TZP in the incisal/occlusal zone along with the elevated flexural strength of 3Y-TZP in the dentin/body zone to boost the stability.^17^

 Translucency is one of the main characteristics of human dentition and is considered a crucial factor of dental restorative aesthetics. TP assesses the variation in light reflection across the specimen among a highly absorbent backdrop (black background) and a highly reflective backdrop (white background).^18^ The previous study demonstrated that the translucency of lithium disilicate was higher than that of 3Y-PSZ and 4Y-PSZ (4 mol% yttria partially stabilized zirconia), although it did not differ significantly from that of 5Y-PSZ.^19^ Color stability is necessary for cosmetic restorations to have long-term clinical success because it guarantees consistent color matching. Nevertheless, aging has an impact on dental ceramics’ translucency and color. It has been demonstrated that artificial aging by autoclave can cause some hydrothermal aging.^20^ Chevalier et al^21^ reported that autoclaving for one hour at 134 °C, in theory, is similar to 3‒4 years in a clinical setting. The esthetic success of new zirconia generations under hydrothermal aging or different oral conditions has not been adequately studied in the literature. As a result, the current study aimed to investigate whether hydrothermal aging affects the color, translucency, and contrast ratio (CR) of the examined materials. Consequently, the present research’s null hypothesis was that hydrothermal aging would not affect the color, translucency, and CR of tested materials (zirconia and lithium disilicate).

## Methods

**Figure 1:**
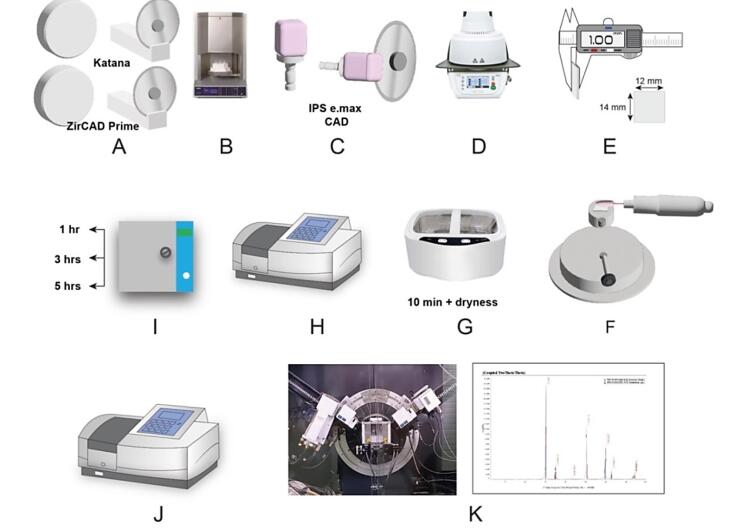
A schematic flow chart of the experimental procedure from A to K. (A) IPS e.max ZirCAD Prime and Katana zirconia preparation by Isomet. (B) Sintering of zirconia samples. (C) IPS e.max samples preparation by Isomet. (D) Crystallization of IPS e.max. (E) Digital calibration of all samples in final dimension (12 × 14 × 1 mm). (F) Polishing of samples using a customized device. (G) Ultrasonic cleaning up to 10 minutes and drying. (H) Spectrophotometer measurement before aging of samples in an autoclave. (I) Class B autoclave for aging times (1, 3, and 5 hours). (J) Spectrophotometer measurement after aging of samples. (K) X-ray diffraction for all samples before and after aging times (1, 3, and 5 hours).

 According to a previous investigation,^22^ the sample size needed for every group was established using Thompson’s power analysis test, with a confidence level of 95%, an α level of 0.05, and a β of 0.1. It was determined that 10 samples were needed for each group. Thirty samples were fabricated and divided into three groups (n = 10): group Z: IPS e.max ZirCAD prime, multilayered gradient zirconia (3Y/5Y-TZP) with highly translucent 5Y-TZP incisal zone, transition zone, 3Y-TZP cervical zone; group K: Katana Ultra Translucent multilayered zirconia UTML (5Y-TZP); and Group E: IPS e.max CAD (lithium disilicate glass-ceramic) (Tables 1 and 2; Figure 1).

**Table 1:** Materials used in the current study

**Material**	**Chemical Composition**	**Manufacturer & Lot Number**
IPS e.max ZirCAD prime (Z)	ZrO_2_ 88.0- 95.5% Y_2_O_3_ > 4.5%- ≤ 7.0% HfO_2_: ≤ 5.0% Al_2_O_3_: ≤ 1.0%, Other oxides: ≤ 1.5%	Ivoclar Vivadent; Schaan, Liechtenstein (Lot. Nr: Z00C8R)
Katana zirconia UTML (K)	ZrO_2_ + HfO_2_: 87‒92% Y_2_O_3_: 8‒11%, Other oxides: 0‒2%	Kurary, Noritake Dental Inc, Miyoshi-cho Miyoshi, Japan. (Lot. Nr: EKUUO)
IPS e.max CAD (E)	80% SiO_2, _19% Li_2_O, 13% K_2_O, 11% P_2_O_5_, 8% ZrO_2_, 5% ZnO, 5% Al_2_O_3_, 8% MgO, coloring oxides 0‒12%	Ivoclar Vivadent; Schaan, Liechtenstein (Lot. Nr: Z019VN)

**Table 2:** Properties of the used materials

**Properties**	**IPS- e.max CAD**	**Katana zirconia (UTML)**	**IPS-e.maxZirCAD Prime**
Flexural strength	360 ± 60 MPa	557 MPa	650‒850 MPa
Modulus of elasticity	95 ± 5 GPa	214‒217 GPa	
Fracture toughness	2.0 - 2.5 MPa.m^1/2^		3.6 MPa. m^1/2^
Vickers hardness	5800 ± 200 MPa	1.280 HV10	23.03 ± 0.56 GPa
Coefficient of thermal expansion	10.15 ± 0.4 10^-6^ K^-1^ (100‒400°C)	9.7 × 10^-6^ K^-1^ (25‒500°C)	10.75 ± 0.25 × 10^-6^ K^-1^ (100‒400°C)

**Figure 2:**
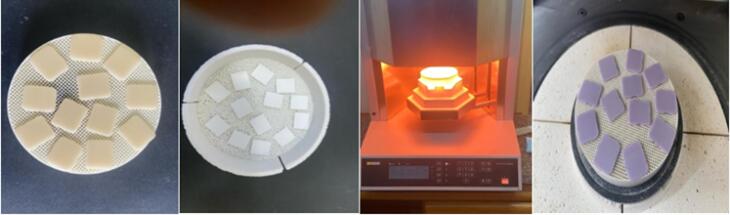
Katana and ZirCAD Prime Zirconia sintering, while e.max ceramic crystallization.

 Thirty rectangular samples with a final dimension of (12 × 14 × 1 mm in thickness)^23^ were sectioned using a low-speed diamond high-precision saw (Isomet 4000, Buehler Ltd, Lake Bluff, LL, USA) at 2500 rpm and under water coolant. Following cutting, a digital caliper (Mitutoyo 200 mm/8 inch, Absolute Digimatic Vernier Caliper, Japan) with an 0.01 mm accuracy was used to verify the samples’ thickness. Zirconia samples were sectioned 20% greater in size to compensate for shrinkage following sintering. Sintering of zirconia samples and crystallization of IPS e.max ceramic samples were achieved according to the manufacturer’s instructions for each type (Figure 2). Zirconia samples (groups Z and K) were sintered in a high-temperature sintering furnace (inFire HTC, Dentsply Sirona) according to the manufacturer’s instructions; group Z was sintered for 2 hours at 1500 °C, and group K was sintered at 1550 °C, with a holding time of 2 hours. IPS e.max CAD samples were crystallized using the Programat P3010 furnace (Programat EP-3010 Furnace, Ivoclar Vivadent) with the specific program for IPS e-max crystallization according to the manufacturer’s instructions (at a temperature of 840 °C and holding time of 7 minutes).

 All the ceramic samples underwent hydrothermal aging using a class B autoclave, as per the guidelines of the International Organization for Standardization (ISO 13356:2015).^24^ The standard conditions involved maintaining a pressure of 0.2 MPa and a temperature of 134 °C for different durations: 0 (control), 1, 3, and 5 hours.^24^ It has been documented that autoclaving at a pressure of 0.2 MPa for 1 hour at 134 °C is comparable to a clinical setting lasting 3‒4 years.^25^

 A spectrophotometer (Cary5000UV-Vis-NIRSpectrophotometer; Agilent Technologies USA.) was employed to determine L*a*b* color coordinates of all the samples regarding the CIEL*a*b* color scale before (baseline) and after hydrothermal aging (after 1, 3, and 5 hours).Before evaluating every sample’s color, the spectrophotometer was calibrated using an integrating sphere connection. L*a*b* coordinates of color were established via the CIEL*a*b* color scale using standard illuminant D65 and 10 degrees standard observer angle over white (L* = 99.85, a* = -0.01, b* = -0.15) and black (L* = 2.06, a* = -0.46, b* = 1.10) backgrounds. The samples were positioned in the middle of the measurement port and kept there for the two foundations, and the spectrophotometer remained in the same position during all the measurements. Within 5-nm periods, the relative reflectivity measurements were acquired in the visible wavelengths: between 380 and 780 nm.

 The color difference (∆E_00_)of each sample was achieved by determining the sample’s color difference compared to a black (L* = 2.06, a* = -0.46, b* = 1.10) background before and after hydrothermal aging (after 1, 3, and 5 hours) using the following equation:^26^

 ΔE_00_ = [(ΔL’/K_L_S_L_)^2^ + (ΔC’/K_C_S_C_)^2^ + (ΔH’/K_H_S_H_)^2^ + R_T_ (ΔC’/K_C_S_C_) (ΔH’/K_H_S_H_)]^1/2^

 The translucency parameter (TP_00_) values were assessed based on CIEDE2000 by measuring the color difference of each sample against black (L* = 2.06, a* = -0.46, b* = 1.10) and white (L* = 99.85, a* = -0.01, b* = -0.15) backgrounds before and after hydrothermal aging (after 1, 3, and 5 hours) using the following equation^27^:

 ΔTP_00_ = [(ΔL’/K_L_S_L_)^2^ + (ΔC’/K_C_S_C_)^2^ + (ΔH’/K_H_S_H_)^2^ + R_T_ (ΔC’/K_C_S_C_) (ΔH’/K_H_S_H_)]^1/2^

 where ΔL’, ΔC’, and ΔH’ are the differences in the lightness, chroma, and hue of a given set of samples, respectively. K_L_, K_C_, and K_H_ are parametric factors used to compensate for the mismatch in the experimental conditions; they were fixed at 1 in the current study. S_L_, S_C_, and S_H_ correspond to the weighting functions for lightness, chroma, and hue, respectively. R_T_ represents the rotation function, which is used to adjust for the interaction between the differences in chroma and hue in the blue region.^27,28^

 The CR was evaluated using the following equation: CR**=**Y_black_**/**Y_white_

 where Y_black_ denotes the CIE tristimulus value of the sample against black (L* = 2.06, a* = -0.46, b* = 1.10) background, and Y_white_ denotes the CIE tristimulus value of the sample against the white (L* = 99.85, a* = -0.01, b* = -0.15) background. High CR values (maximum of 1) are associated with opaque objects, while high values of TP are associated with translucent materials.

###  X-ray diffraction 

 X-ray diffraction (XRD, D8 Discover Plus, Burker, Germany)is a widely used method for figuring out a material’s crystalline makeup or composition. The levels of ceramics’ chemical components were assessed via wavelength dispersive x-ray fluorescence spectroscopy (WD-XRF; Bruker S8 Tiger, Germany) employing a 4 KW x-ray tube. XRD was accomplished for all the samples before and after different aging times (1, 3, and 5 hours.). Quant-Express software (Bruker, Karlsruhe, Germany) was used to automatically deliver the raw data on the chemical components of the samples.

###  Statistical analysis

 The mean and standard deviation (SD) values were used to display numerical data. They were analyzed for normality and variance homogeneity by viewing the data distribution and using Shapiro-Wilk and Levene’s tests, respectively. The data were normally distributed, had homogenous variances, and were analyzed using two-way mixed-model ANOVA. A significant threshold of *P* < 0.05 was applied. R statistical analysis version 4.3.3 for Windows was used to analyze the statistics.

## Results

###  Color difference (ΔE_00_)

**Figure 3:**
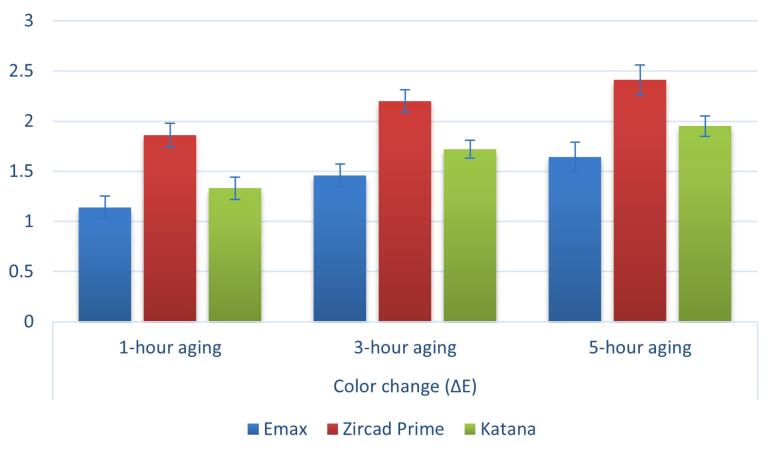
A column chart displaying mean color change (ΔE_00_) values for various materials and aging times

 Both material type and aging time significantly affected ΔE_00_ (*P* < 0.001), whereas the impact of the interaction between them was not statistically significant (*P* = 0.637) (Table 3, Figure 3).

**Table 3:** Effect of different variables and their interactions on color difference (ΔE00)

**Variable**	**Sum of squares (II)**	**df**	**Mean square**	**f-value**	***P*** ** value**
Material	8.64	2	4.32	233.69	< 0.001*
Aging time	4.77	2	2.38	194.28	< 0.001*
Material × aging time	0.03	4	0.01	0.64	0.637ns

df = degree of freedom*; significant (*P* < 0.05) ns; non-significant (*P* ≥ 0.05).

 The various materials differed significantly from one another (*P* < 0.001). After different aging times (1, 3, and 5 hours), the highest value was recorded in ZirCAD Prime (1.86 ± 0.12, 2.20 ± 0.11, and 2.41 ± 0.15, respectively), followed by Katana (1.33 ± 0.11, 1.72 ± 0.09, 1.95 ± 0.10, respectively). In contrast, the lowest value was recorded with IPS e.max (1.14 ± 0.11, 1.46 ± 0.11, and 1.64 ± 0.15, respectively) (Table 4).

**Table 4:** Intergroup and intragroup comparisons; mean and standard deviation (SD) values of color differences (ΔE_00_) for different materials and aging times

**Aging time**	**Color difference (ΔE**_00_**) (Mean±SD)**			***P*** ** value**
	**IPS e.max CAD**	**ZirCAD Prime**	**Katana UTML**	
1-hour aging	1.14 ± 0.11^Cc^	1.86 ± 0.12^Ac^	1.33 ± 0.11^Bc^	< 0.001*
3-hour aging	1.46 ± 0.11^Cb^	2.20 ± 0.11^Ab^	1.72 ± 0.09^Bb^	< 0.001*
5-hour aging	1.64 ± 0.15^Ca^	2.41 ± 0.15^Aa^	1.95 ± 0.10^Ba^	< 0.001*
*P* value	< 0.001*	< 0.001*	< 0.001*	

Different uppercase superscripts indicate a statistically significant difference within the same horizontal row. Different lowercase superscripts indicate a statistically significant difference within the same vertical column. * Significant (*P* < 0.05), ns: non-significant (*P* ≥ 0.05).

 After 1, 3, and 5 hours, a statistically significant difference (*P* < 0.001) was observed across the ΔE_00_ of ceramic types. The highest value was recorded after 5 hours of aging, followed by 3-hour aging, whereas the lowest value was recorded after 1 hour of aging. Every pairwise comparison made after the fact was statistically significant (*P* < 0.001).

###  Translucency parameter (TP_00_)

**Figure 4:**
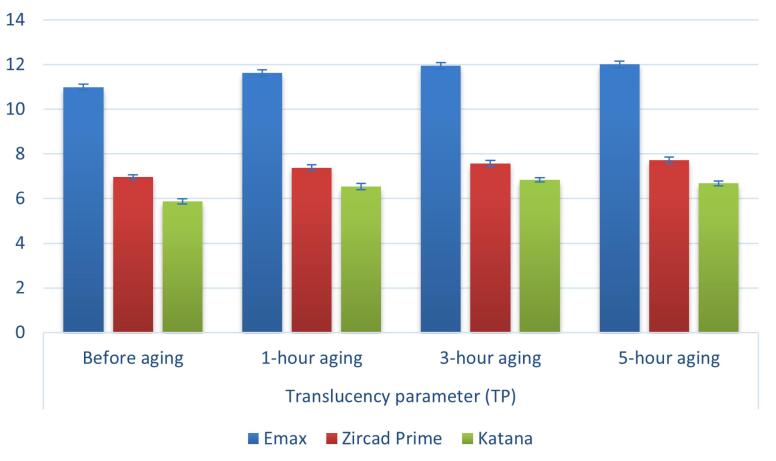
A column chart displaying mean translucency parameter (TP_00_) values for various materials and aging times

 The findings indicated that the mean TP_00_ was significantly impacted (*P* < 0.001) by both ceramic type (independent of time) and aging time (independent of the ceramic type) (Table 5, Figure 4).

**Table 5:** Effect of different variables and their interactions on translucency parameter (TP_00_)

**Variable**	**Sum of Squares (II)**	**df**	**Mean Square**	**f-value**	***P*** ** value**
Material	606.41	2	303.21	7386.82	< 0.001*
Aging time	14.47	2	6.73	526.96	< 0.001*
Material × aging time	0.62	4	0.14	11.33	< 0.001*

df = degree of freedom. * Significant (*P* < 0.05), ns: non-significant (*P* ≥ 0.05).

 The various materials differed significantly from one another (*P* < 0.001). Along the different aging times (1, 3, and 5 hours), the greatest translucency was discovered in IPS e.max (11.63 ± 0.14, 11.94 ± 0.14, and 12.01 ± 0.15, respectively), followed by ZirCAD Prime (7.38 ± 0.13, 7.57 ± 0.14, and 7.71 ± 0.15, respectively). In contrast, the least translucency was discovered at Katana (6.53 ± 0.14, 6.83 ± 0.10, and 6.68 ± 0.11, respectively). Every pairwise comparison made after the fact was statistically significant (*P* < 0.001). Moreover, a statistically significant variation was displayed in the TP_00_ of ceramic types at baseline (before aging) and after 1, 3, and 5 hours. Post hoc pairwise comparisons showed the highest translucency after 5 hours of aging, followed by 3-hour aging, while the lowest translucency was related to before aging (Table 6).

**Table 6:** The repeated measurements’ mean, standard deviation (SD), and outcomes ANOVA test for TP00 comparisons of different ceramic materials and periods

**Aging time**	**Translucency parameter (TP**_00_**) (Mean±SD)**			***P*** ** value**
	**IPS e.max CAD**	**ZirCAD Prime**	**Katana UTML**	
Before aging	10.99 ± 0.13^Ac^	6.96 ± 0.11^Bd^	5.87 ± 0.11^Cc^	< 0.001*
1-hour aging	11.63 ± 0.14^Ab^	7.38 ± 0.13^Bc^	6.53 ± 0.14^Cb^	< 0.001*
3-hour aging	11.94 ± 0.14^Aa^	7.57 ± 0.14^Bb^	6.83 ± 0.10^Ca^	< 0.001*
5-hour aging	12.01 ± 0.15^Aa^	7.71 ± 0.15^Ba^	6.68 ± 0.11^Cb^	< 0.001*
*P* value	< 0.001*	< 0.001*	< 0.001*	

Different uppercase superscripts indicate a statistically significant difference within the same horizontal row. Different lowercase superscripts indicate a statistically significant difference within the same vertical column. * Significant (*P* < 0.05), ns: non-significant (*P* ≥ 0.05).

###  Contrast ratio 

 There was a significant interaction between both tested variables (*P* < 0.001) (Table 7). For the IPS e.max group, the highest CR was found before aging (0.69 ± 0.00), followed by 1-hour aging (0.67 ± 0.00) and 3-hour aging (0.67 ± 0.00), while the lowest CR was found in 5-hour aging (0.66 ± 0.00). For the ZirCAD Prime group, the highest CR was found before aging (0.78 ± 0.00), followed by 1-hour aging (0.76 ± 0.00) and 3-hour aging (0.75 ± 0.01), while the lowest CR was found in 5-hour aging (0.75 ± 0.01). For the Katana group, the highest CR was found before aging (0.80 ± 0.00), followed by 1-hour aging (0.77 ± 0.01) and 5-hour aging (0.77 ± 0.00), while the lowest CR was found in 3-hour aging (0.76 ± 0.00). Post hoc pairwise comparisons showed a significantly higher CR before aging compared to other aging times (*P* < 0.001) (Table 8).

**Table 7:** Effect of different variables and their interactions on contrast ratio (CR)

**Variable**	**Sum of squares (II)**	**df**	**Mean square**	**f-value**	***P*** ** value**
Material	0.24	2	0.12	2885.92	< 0.001*
Aging time	0.01	2	0.01	308.32	< 0.001*
Material × aging time	0.00	4	0.00	9.76	< 0.001*

df = degree of freedom. * Significant (*P* < 0.05), ns: non-significant (*P* ≥ 0.05).

**Table 8:** Intergroup and intragroup comparisons; mean and standard deviation (SD) values of contrast ratio (CR) for various materials and aging times

**Aging time**	**Contrast ratio (CR) (Mean±SD)**			***P*** ** value**
	**IPS e.max CAD**	**ZirCAD Prime**	**Katana UTML**	
Before aging	0.69 ± 0.00^Ca^	0.78 ± 0.00^Ba^	0.80 ± 0.00^Aa^	< 0.001*
1-hour aging	0.67 ± 0.00^Cb^	0.76 ± 0.00^Bb^	0.77 ± 0.01^Ab^	< 0.001*
3-hour aging	0.67 ± 0.00^Cc^	0.75 ± 0.01^Bbc^	0.76 ± 0.00^Ac^	< 0.001*
5-hour aging	0.66 ± 0.00^Cc^	0.75 ± 0.01^Bc^	0.77 ± 0.00^Ab^	< 0.001*
P value	< 0.001*	< 0.001*	< 0.001*	

Different uppercase superscripts indicate a statistically significant difference within the same horizontal row. Different lowercase superscripts indicate a statistically significant difference within the same vertical column. * Significant (*P* < 0.05), ns: non-significant (*P* ≥ 0.05).

###  X-ray diffraction 

 Regarding XRD results, aging times resulted in tetragonal (t)/monoclinic (m) phase transformation in group Z (ZirCAD Prime, gradient zirconia)) only. However, no monoclinic phase transformation was detected in the 5Y (Katana), group K. For group E (IPS e.max CAD), composed mainly of Li_2_(Si_2_O_5_) and Li_2_(Si_3_O_7_) phases at different ratios, the main phase is Li_2_(Si_2_O_5_) crystalline phase in the material, which increased with increased aging time. Table 9 presents the crystal content of each material and crystal peak readings.

**Table 9:** XRD demonstrating crystalline content (%) for each material after different aging times

**Group**	**ZirCAD Prime**				**Katana UTML**			
	**Control**	**1 hour**	**3 hours**	**5 hours**	**Control**	**1 hour**	**3 hours**	**5 hours**
Tetragonal %	66.2	51	47.9	53.7	60.3	61.1	58.1	57.5
Cubic %	33.8	28.5	29.2	30.4	39.7	38.9	41.9	42.5
Monoclinic %	0	20.5	22.9	15.9	0	0	0	0
	**IPS e.max CAD**							
	**Control**	**1 hour**	**3 hours**	**5 hours**				
Dilithium disilicate%	66.4	65.5	77	80.5				
Dilithium closoheptaoxotrisilicate%	33.6	34.5	23	19.5				

## Discussion

 An all-ceramic restoration that looks natural must be similar in color and translucency to natural teeth. Without veneering, lithium disilicate ceramics are appropriate for anterior and posterior crowns, thanks to their high optical properties and reasonable mechanical ones.^29^ The production of zirconia for dental prosthetic restorations has expanded during the last few years. Adding yttria to zirconia creates more cubic phases, enhancing translucency. Nevertheless, some tetragonal phases decrease the strength.^12^ Based on the current research’s findings, the null hypothesis was rejected as hydrothermal aging significantly impacted the ceramic samples’ optical characteristics.Analytical tools can be used to quantify color to obtain more accurate measurements of color.^30^ In the current research, the coordination of colors in CIE_00 _L*a*b* was evaluated using a spectrophotometer on flat ceramic samples (12 × 14 × 1 mm). Since this device might be less precise when evaluating a curved surface, this situation was irrelevant to the current investigation. Other multiple investigations confirmed the accuracy and validity of spectrophotometer in color measurement.^31-33^

 Hydrothermal aging is the most popular technique for accelerating age; however, exposure to UV radiation and using water spray in a weathering device might be used, too.^34,35^ Hydrothermal aging in autoclaves is one of the most widely recognized techniques for simulating clinical settings. When portions of a material’s tetragonal zirconia phase are subjected to moisture or hydrothermal degradation, this might change the mechanical and optical characteristics of the zirconia subsurface and cause a t/m transition.^36^ In this research, the zirconia samples underwent polishing treatments, which are advised as a crucial clinical process to improve sample outcomes. According to Pereira et al,^37^ smoother surfaces may have less area for water interactions, making them less susceptible to hydrothermal aging. A 1-mm ceramic thickness was used as the standard recommended for monolithic zirconia restorations. According to Tabatabaian et al,^23^ a minimum thickness of 1 and 1.6 mm of zirconia coping is needed to hide the material to obtain appropriate tolerance and observable tolerance threshold correspondingly.

 Various investigations have documented a color change in dental ceramics due to aging.^9,38^ In general, numerous variables influence the color of ceramic materials, including surface cracks, roughness, and method of sintering. Therefore, when temperature time increases, grain size increases, and porosity is reduced, the formation of a highly organized crystalline structure that permits light reflection might be the main factor affecting the color difference. Theoretically, thermal conditions may influence the coloring pigments that were put into Y-TZP ceramics, which led to pigment disintegration and instability of color.^39^ Nakamura et al^14^ demonstrated that, following autoclaving for 100 hours, ΔE values were under the perceptibility threshold despite LTD’s impact on the color characteristics of 3Y-TZP. Additionally, they discovered that the colored 3Y-TZP had a larger cubic fraction and greater quantities of trivalent dopants (Fe_2_O_3_ and Er_2_O_3_) than the noncolored zirconia, which resulted in higher resistance to LTD.

 The color difference (ΔE_00_) of samples was assessed through perceptibility threshold “PT” of 50:50% (ΔE_00_ = 0.8) and acceptability threshold “AT” of 50:50% (ΔE_00_ = 1.8) according to Paravina et al.^26^ Greater ΔE_00_ value more than (0.8) (PT) is considered clinically perceptible, and a ΔE_00_ value higher than 1.8 (AT) is considered clinically unacceptable. While Salas et al^27^ determined the translucency perceptibility threshold (TPT00) = 0.62 units and the translucency acceptability threshold (TAT00) = 2.62 units.

 All color measurements were significantly different in the present research regardless of ceramic type or aging time. Group Z showed the highest mean ΔE_00_, followed by groups K and E. When exposed to ambient temperature and humidity, stabilized zirconia polycrystals undergo a degradation process known as low-temperature degradation (LTD) aging, during which stabilizing oxides are removed from the substance’s structure. This results in a crack, damage to structural integrity, and a reduction in the mechanical characteristics of the material by causing the tetragonal-to-monoclinic phase transformation. Furthermore, the alumina concentration was reduced in monolithic zirconia to increase translucency. The resistance to deterioration at low temperatures is ascribed to alumina. Consequently, monolithic zirconia can be more vulnerable to LTD.^40^

 In the present research, with increasing aging time, a significant color change of the ceramic sample (ΔE_00_) was noted, which was still within the clinically acceptable range, except for group Z after 3 and 5 hours and group K after 5 hours. Various studies have shown that following artificially accelerated aging, zirconia had a much greater color shift than lithium disilicate.^41,42^ Kim et al^42^ showed that after autoclave-based artificial aging at 134 °C at 0.2 MPa for 0, 1, 3, 5, or 10 hours, Katana monolithic zirconia displayed a significantly greater color change than IPS e.max CAD, which might be attributed to the low a* and b* values and high L* values. Unlike the current outcomes, Subaşı et al^43^ showed non-significant variations in the color change values between three CAD-CAM monolithic ceramic materials. Following 5000 thermocycles in a coffee solution, the three materials (Vita Suprinity PC, IPS e.max CAD, and InCoris TZI C) had identical thicknesses. Inconsistencies in research findings may be due to the differences in sample preparation and aging techniques.

 Translucency is one of the main determining elements and an important one when choosing. The present study documented an increase in TP and a reduction in CR. Group E was the most translucent with the lowest CR, which concurs with many earlier examinations.^4,5,7,44^ This may be explained by the lithium disilicate glass content. When evaluated at the identical thickness, the translucency of recent types of monolithic zirconia remains less than that of lithium disilicate, although a significant enhancement in comparison to traditional ones.^44,45^

 The findings of the present research agreed with Kim et al,^42^ who evaluated the impact of hydrothermal aging on the optical characteristics of pre-colored monolithic zirconia ceramics and demonstrated that the aging by hydrothermal means impacted its optical qualities and the translucency increased with a longer aging period. Additionally, with longer aging times, greater TP values might be obtained due to particular metal oxides in coloring pigments, promoting the microstructure-level production of cubic zirconia. However, according to the findings of the XRD of the present research, there was no increase in cubic zirconia after aging time, so these changes were attributed to other factors such as the aging process, roughness, and porosity.

 Since various zirconia materials include varying concentrations of Y_2_O_3_, Al_2_O_3_, and other oxides, they display varying translucencies. As dopants, metal oxides disperse incident rays and reflect light. Perhaps increased translucency results from the dopant separation of trivalent ions to the grain boundaries, which boosts hydrothermal stability.^46^ Zhang^47^ introduced 0.2% mole La_2_O_3_ in traditional Al_2_O_3_-doped 3Y-TZP, yielding good mechanical characteristics, lack of LTD, and translucency similar to lithium disilicate. This co-doping of La_2_O_3_ and Al_2_O_3_ caused the whole dissolving of La_2_O_3_ in the zirconia grains devoid of the formation of a secondary phase, which is what gave the material its increased translucency and resistance to LTD. However, these results did not agree with Ahmed et al,^48^ who assessed the influence of artificial aging on the ultra-translucent tetragonal/cubic zirconia’s optical characteristics (53% cubic and 47% tetragonal). They demonstrated no significant change in the translucency after rapid autoclave treatment for one or three hours. Their results were supported by the significant quantity of cubic phase, which renders the zirconia stable in hydrothermal conditions. Volpato et al^49^ also assessed the color and translucency of zirconia that had been artificially aged in a steam autoclave for four hours and showed no alteration in translucency in any of the samples, either before or after a coloring liquid was applied.

 CR has been used to assess a material’s opacity. TP is more commonly used in literature than CR. Spink et al^50^ stated that CR, an indicator of diffuse reflectance, cannot identify minute variations in light transmission if materials have large scattering and absorption coefficients. Samples with more TP values and less CR values exhibit more translucency or less opacity.^51^

 The ISO standard 13 356:2008 states that for appropriate biomedical usage, the maximum quantity of Y-TZP monoclinic phase following autoclaving and aging at 134°C under 0.2 MPa for five hours should not exceed 25%.^24^ In the present investigation, group Z showed greater t/m transition behavior as the aging period increased, causing 20.5% of m-phase at 1 hour and 22.9% at 3 hours of artificial aging. Quantification of crystalline structures is commonly achieved by employing the XRD technique. Crystal peak readings may be used to readily define the phenomena of t/m transformation and alterations in the lattice position against the transport of atoms.^52^

 Regarding XRD results, aging times caused the phase transformation between tetragonal (t) and monoclinic (m) for the (ZirCAD Prime) group Z only, which explains the quantity of yttria and how it relates to toughening transformation. Generally, zirconia with less yttria has more tetragonal particles that can change into monoclinic. However, there was no formation of monoclinic phase transformation in the 5Y (Katana) group K. Due to the high yttria concentration, 5Y zirconia has a high stability, which might account for the absence of phase transformation. At the same time, in group E (IPS e.max), composed of Li_2_(Si_2_O_5_) and Li_2_ (Si_3_O_7_) phases at different ratios, the main phase is the Li_2_(Si_2_O_5_) crystalline phase in the material, which increased with increased aging time.The limitation of the present research was that samples used only one thickness for all types of ceramics under one type of aging. However, dental restorations in the oral environment are subjected to numerous stimuli, such as mechanical aging and pH fluctuations. Further research is thus needed to evaluate the alterations in the optical characteristics of dental ceramics considering different aging circumstances to represent the clinical setting.

## Conclusion

 Considering the constraints of the present research, the subsequent conclusions can be noted:

1. All the ceramic groups displayed a significant change in the optical properties following hydrothermal aging.
2. Translucency increased with increasing hydrothermal aging time.
3. ZirCAD Prime gradient zirconia was more translucent with aging than the Katana type.

## Competing Interests

 The authors declared no conflicts of interest.

## Ethics Approval

 The research proposal was approved by Research Ethics Committee of the Faculty of Dental Medicine, Al-Azhar University, Cairo, Egypt (No: 578/2600).
